# Serum Antioxidant Capacity Predicts Prognosis in Patients with Metastatic Colorectal Cancer: An Original Cohort Study

**DOI:** 10.3390/antiox15050595

**Published:** 2026-05-08

**Authors:** Katsuji Sawai, Nobuhiro Maegawa, Kenji Koneri, Takanori Goi

**Affiliations:** First Department of Surgery, University of Fukui, Fukui 910-1193, Japan; maechan@u-fukui.ac.jp (N.M.); koneri@u-fukui.ac.jp (K.K.); tgoi@u-fukui.ac.jp (T.G.)

**Keywords:** colorectal cancer, oxidative stress, antioxidant capacity, chemotherapy resistance, biological antioxidant potential

## Abstract

Reactive oxygen species contribute to the cytotoxic effects of anticancer drugs; however, the clinical relevance of systemic antioxidant capacity in metastatic colorectal cancer (CRC) remains unclear. In this original single-center observational cohort study, we examined the association of baseline blood antioxidant capacity with chemotherapy response and prognosis in 84 patients with stage IV CRC who underwent primary tumor resection followed by systemic chemotherapy between 2015 and 2020. Baseline antioxidant capacity was assessed preoperatively using biological antioxidant potential (BAP) assays. Chemotherapy response was evaluated using contrast-enhanced computed tomography at 4 months using Response Evaluation Criteria in Solid Tumors v1.1. Three-year disease-specific survival (DSS) was assessed. Associations with treatment response were analyzed using linear regression. Survival outcomes were evaluated using Kaplan–Meier and Cox proportional hazards models. Baseline BAP was significantly associated with poorer chemotherapy response; higher BAP levels predicted greater treatment resistance in multivariable analysis (*p* = 0.009). Kaplan–Meier analysis demonstrated significantly worse 3-year DSS in the high-BAP group than in the low-BAP group (35.6% vs. 55.5%, log-rank *p* = 0.019). In multivariate Cox regression analysis, high BAP independently predicted poor DSS (hazard ratio 2.174, 95% confidence interval 1.103–4.283, *p* = 0.009). Elevated baseline systemic antioxidant capacity was associated with reduced chemotherapy effectiveness and poorer DSS in patients with stage IV CRC.

## 1. Introduction

Colorectal cancer (CRC) ranks as the third most frequently diagnosed cancer and is the second leading cause of cancer-related mortality worldwide, with around 1.9 million new cases and over 900,000 deaths occurring globally each year [1]. Despite advancements in screening and multimodal therapy, about 20% of patients are found to have metastatic disease at the time of diagnosis, and an additional 20–30% develop distant metastases after undergoing treatment intended to be curative [2].

Metastatic colorectal cancer (mCRC) is primarily treated with systemic chemotherapy. Population-based cohorts have reported a median overall survival of approximately 25–35 months [3]. However, the therapeutic benefit varies widely among individuals, and reliable biomarkers that predict treatment response remain limited in routine clinical practice.

Reactive oxygen species (ROS) and antioxidant defenses coexist in a dynamic balance and exert context-dependent biological effects. In normal cells, ROS are signaling molecules that regulate metabolism and proliferation, modulate enzymatic and transcriptional activity, and shape immune responses [4,5,6,7]. However, excessive ROS can oxidatively damage lipids, proteins, and nucleic acids, including DNA, thereby contributing to carcinogenesis and malignant progression [8,9,10]. Nuclear factor erythroid 2-related factor 2 (NRF2) is a key regulator of cellular reduction–oxidation (redox) homeostasis. Upon sensing oxidative stress, NRF2 induces a transcriptional antioxidant program that supports intracellular redox balance by enhancing glutathione (GSH) synthesis and thioredoxin (TRX) pathways and upregulating antioxidant enzymes, including superoxide dismutase (SOD), catalase (CAT), and glutathione peroxidase, to limit pathological ROS accumulation [4,10,11].

Cancer tissues often exhibit higher ROS levels than those in normal tissues, driven by their increased metabolic activity, mitochondrial dysfunction, and inflammatory cues [5,11,12]. Elevated ROS can activate signaling cascades that support tumor cell proliferation and survival and stimulate angiogenic factors, including vascular endothelial growth factor (VEGF), thereby promoting tumor angiogenesis and contributing to the tumor microenvironment. ROS also participate in epithelial–mesenchymal transition, which can increase invasion, migration, and metastatic potential [4,13,14]. Conversely, when oxidative stress surpasses a certain limit, tumor cells might be unable to adjust, resulting in the initiation of cell death mechanisms such as apoptosis, necrosis, and ferroptosis, which is a form of regulated cell death dependent on iron, potentially leading to tumor reduction [8,15,16].

This dual role of ROS is clinically relevant because several cytotoxic agents used to treat mCRC can increase intracellular ROS levels and, at least in part, contribute to DNA damage, mitochondrial dysfunction, and apoptosis [17,18]. Among these, oxaliplatin promotes mitochondrial ROS accumulation and oxidative DNA injury, which can amplify apoptotic signaling [19]. Fluoropyrimidines can also enhance oxidative stress through mitochondrial dysfunction and impaired regeneration of antioxidant molecules [20]. In parallel, tumor cells can adapt to chronic oxidative pressure by augmenting antioxidant defenses, including GSH synthesis, the TRX pathway, peroxiredoxins, and NRF2-driven programs [11,21]. Experimental models further suggest that exogenous antioxidants can attenuate chemotherapy-induced tumor cell death, that NRF2 activation increases the expression of GSH-related enzymes and supports survival under cytotoxic stress, and that GSH depletion can enhance chemosensitivity [22,23,24,25,26,27]. Collectively, these findings suggest that antioxidant capacity can modulate treatment efficacy.

Antioxidant defenses contribute to cellular homeostasis and limit oxidative damage in normal tissues and are, therefore, generally considered beneficial in healthy individuals. However, in settings where the therapeutic efficacy of chemotherapy may depend, at least in part, on the induction of oxidative stress, a higher systemic or tumor-associated antioxidant capacity could counteract ROS-mediated cytotoxicity, leading to attenuated treatment effects. Thus, the clinical implications of “antioxidant capacity” may differ between physiological conditions and the context of anticancer chemotherapy.

Despite these mechanistic links, clinical data directly testing whether pretreatment circulating antioxidant capacity predicts chemotherapy response or prognosis in mCRC are scarce. Therefore, we hypothesized that higher systemic antioxidant capacity before treatment is associated with a poorer radiologic response and inferior disease-specific survival (DSS). To evaluate this hypothesis, we analyzed data from patients with mCRC who underwent primary tumor resection at our institution between 2015 and 2020 and subsequently received systemic chemotherapy. We assessed pretreatment antioxidant capacity using the biological antioxidant potential (BAP) assay and examined its association with treatment response based on computed tomography (CT) at 4 months and DSS.

## 2. Materials and Methods

### 2.1. Study Design and Patient Population

In this single-center observational cohort study, we retrospectively reviewed data from consecutive patients treated at our institution between January 2015 and December 2020. This cohort included 84 patients with stage IV CRC who underwent primary tumor resection followed by systemic chemotherapy.

The eligibility criteria were: (1) histologically confirmed colorectal adenocarcinoma; (2) distant metastasis present at the time of surgery, corresponding to stage IV disease according to the TNM Classification of Malignant Tumors (8th edition); (3) receipt of systemic chemotherapy after primary tumor resection; (4) availability of preoperative serum samples for redox biomarker analysis; and (5) adequate clinical, laboratory, imaging, and follow-up data for evaluation.

The exclusion criteria were patient conditions, including synchronous or metachronous malignancies, inflammatory bowel disease, immunosuppressive disorders, severe comorbid illnesses, or hemodialysis.

We extracted clinical data from institutional medical records, including age, sex, tumor size, depth of invasion, lymph node status, number of metastatic organs, preoperative chemotherapy, treatment regimens, and DSS.

The patients were followed up through outpatient visits and imaging studies.

This study was conducted in accordance with the principles embodied in the Declaration of Helsinki and was approved by the Ethics Committee of Fukui University Hospital (Approval No. 20200058, 1 April 2017). Informed consent was obtained from all subjects involved in the study.

### 2.2. Measurement of Blood Oxidative Stress and Antioxidant Capacity

Peripheral venous blood samples were collected before primary tumor resection. Serum was separated via centrifugation and stored at −80 °C until analysis.

To evaluate systemic oxidative stress, the derivatives of reactive oxygen metabolite (d-ROM) test was utilized, while the BAP test was employed to measure systemic antioxidant capacity. Both tests were conducted using a Free Radical Elective Evaluator system (FREE Carpe Diem; Wismerll Co., Ltd., Tokyo, Japan), which features a spectrophotometric device. The reader and specific reagents were tailored for the FREE Carpe Diem system and were applied following the manufacturer’s guidelines.

In the d-ROM test, a mixture of 20 μL of serum and 1 mL of buffered solution was prepared in a cuvette, to which 20 μL of chromogenic substrate was added. After ensuring the mixture was well combined, the cuvette was placed in the analyzer’s thermostatic block for a 5 min incubation at 37 °C. The absorbance was subsequently recorded at a wavelength of 505 nm. Results were reported in arbitrary units (U.CARR), where 1 U.CARR equates to 0.8 mg/L of hydrogen peroxide. The normal range was set between 250 and 300 U.CARR, with readings of 300 U.CARR or higher indicating elevated serum oxidative stress, signifying an overproduction of free radicals [28,29,30,31,32].

In the BAP test, 10 μL of serum is combined with 1 mL of the assay mixture, and the reduction of ferric (Fe^3+^) iron is measured over a 5 min period, expressed in μmol/L. When FeCl3 is introduced to a colorless solution containing a chelating acid derivative, the solution turns red due to the presence of Fe^3+^ ions. This red color fades as Fe^3+^ ions are converted to ferrous (Fe^2+^) ions by the plasma’s antioxidant activity. The extent of this color change is assessed using spectrophotometry to determine antioxidant capacity. In healthy individuals, typical BAP values exceed 2200 μmol/L [31,32].

We examined the associations of d-ROM and BAP levels with clinicopathological factors using these preoperative measurements. We also evaluated the relationship between preoperative d-ROM and BAP levels.

### 2.3. Assessment of Treatment Response

The effectiveness of the initial chemotherapy was assessed 4 months post-surgery using contrast-enhanced CT. Tumor response was measured according to the Response Evaluation Criteria in Solid Tumors (RECIST) version 1.1. This included partial response (PR), defined as a ≥50% reduction in the overall tumor size; stable disease (SD), defined as a decrease of <50% or an increase of <25%; and progressive disease (PD), indicated by a tumor size increase of ≥25% or the appearance of new lesions [33]. No patients in this cohort showed a complete response (CR).

For quantitative analysis, treatment response was converted into an ordinal score to allow regression modeling (PR = 1, SD = 2, and PD = 3), reflecting increasing levels of treatment resistance.

### 2.4. Survival Outcome

The primary survival endpoint was DSS, defined as the time from primary tumor resection to CRC-attributable death. Patients who were alive at the end of the follow-up or who died of non-cancer-related causes were censored at the date of last contact.

Patients were monitored for up to 3 years following surgery. Kaplan–Meier survival curves were created for DSS after categorizing patients into high- and low-risk groups based on d-ROM and BAP levels. The optimal cutoff values for d-ROMs and BAP in predicting the 3-year DSS were identified through receiver operating characteristic (ROC) curve analysis. ROC analyses for predicting the 3-year DSS showed areas under the curve (AUCs) of 0.559 for d-ROMs (cutoff, 458 U.CARR; sensitivity, 42.8%; specificity, 74.0%) and 0.509 for BAP (cutoff, 2637 μmol/L; sensitivity, 40.7%; specificity, 76.7%) (Figures S1 and S2). The ROC-derived cutoff should be interpreted with caution because the AUC indicated limited discriminatory ability. Therefore, we additionally performed a sensitivity analysis using the median BAP value as the cutoff.

### 2.5. Statistical Analysis

We summarized continuous variables using medians and interquartile ranges.

Correlations between d-ROMs and BAP were evaluated using Spearman’s rank correlation coefficients. We examined associations between redox biomarkers and clinicopathological variables using appropriate nonparametric tests. Differences in treatment response were analyzed using the Mann–Whitney U test. Finally, we performed linear regression analysis to assess independent associations between redox biomarker levels and therapeutic efficacy, while adjusting for relevant clinical variables.

For survival analysis, we compared DSS curves using the log-rank test and constructed multivariate Cox proportional hazards regression models to identify independent prognostic factors for the 3-year DSS. Hazard ratios (HRs) and 95% confidence intervals (CIs) were calculated.

Statistical analyses were conducted using IBM SPSS Statistics for Windows, version 21.0 (IBM Japan, Ltd., Tokyo, Japan). All tests were two-tailed, with statistical significance determined at *p* < 0.05.

## 3. Results

### 3.1. Patient Characteristics and Distribution of Redox Biomarkers

The analysis included 84 patients with stage IV CRC who underwent primary tumor resection followed by systemic chemotherapy. Patient age ranged from 38 to 85 years (median, 67 years). Tumor diameter ranged from 13 to 115 mm (median, 50 mm). Tumor depth, according to the TNM classification, was T2 in two patients, T3 in 15, T4a in 55, and T4b in 12. The number of metastatic organs was one in 54 patients, two in 25, and three in five. Liver, peritoneal, and lung metastases were present in 61, 18, and 25 patients, respectively.

Preoperative treatment was administered to 12 patients and included chemoradiotherapy in two patients; molecular targeted therapy (panitumumab or bevacizumab) combined with a doublet regimen in eight patients (modified FOLFOX6 [mFOLFOX6], consisting of oxaliplatin, leucovorin, and fluorouracil, or FOLFIRI, consisting of irinotecan, leucovorin, and fluorouracil); and molecular targeted therapy combined with a triplet regimen in two patients (FOLFOXIRI, consisting of fluorouracil [5-FU], leucovorin, oxaliplatin, and irinotecan).

Surgical procedures included colectomy (44 patients), high anterior resection (12 patients), low anterior resection (23 patients), and abdominoperineal resection (five patients).

**Figure 1:**
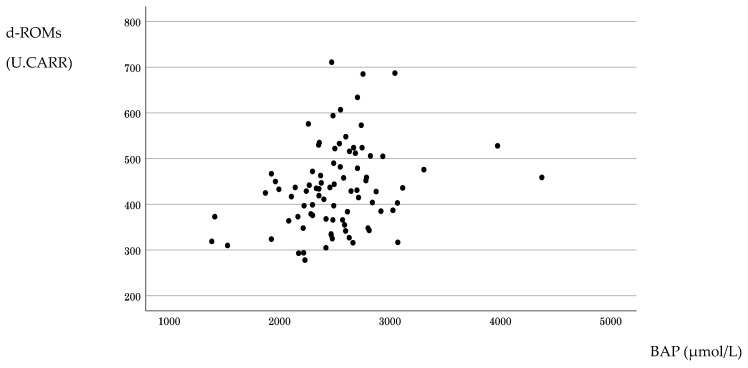
Distribution and correlation of systemic redox biomarkers before treatment. The distributions of derivatives of reactive oxygen metabolite (d-ROM) and biological antioxidant potential (BAP) values measured before surgery and chemotherapy in patients with stage IV colorectal cancer are shown, together with their correlation. Spearman’s rank correlation analysis showed a weakly positive correlation, suggesting that oxidative burden and antioxidant capacity were largely independent parameters in this cohort.

Baseline measurements of systemic oxidative stress and antioxidant capacity obtained before primary tumor resection showed that the distribution of d-ROM values approximated a normal distribution, whereas that for BAP values showed a non-normal distribution. No strong association was observed between d-ROMs and BAP; rather, the correlation was weakly positive (Spearman’s r = 0.294, *p* = 0.007) (Figure 1).

### 3.2. Association Between Redox Biomarkers and Clinicopathological Factors

The associations between preoperative redox markers and clinicopathological factors are summarized in Table 1. d-ROM levels were higher in patients with tumor size ≥5 cm than in those with tumor size <5 cm (*p* = 0.046). No other clinicopathological factor was significantly associated with d-ROM levels. BAP was not significantly associated with the evaluated clinicopathological factors (Table 1).

### 3.3. Treatment Response and Association with Redox Biomarkers

Therapeutic response to first-line postoperative chemotherapy was assessed using contrast-enhanced CT 4 months postoperatively. Ten patients who underwent postoperative chemotherapy received an oral 5-FU agent. A doublet regimen was administered to 74 patients, including 36 who also received additional molecular targeted therapy. Overall, 18 patients achieved a PR, 35 had SD, and 31 had PD. No patients in our cohort experienced CR. In univariate analyses, patients with PD had significantly higher BAP levels than did patients who achieved PR or SD. In contrast, d-ROM levels were not significantly associated with chemotherapy response. No other factors showed a significant association with the therapeutic response (Table 2).

### 3.4. Multivariate Analysis of Therapeutic Response

**Figure 2:**
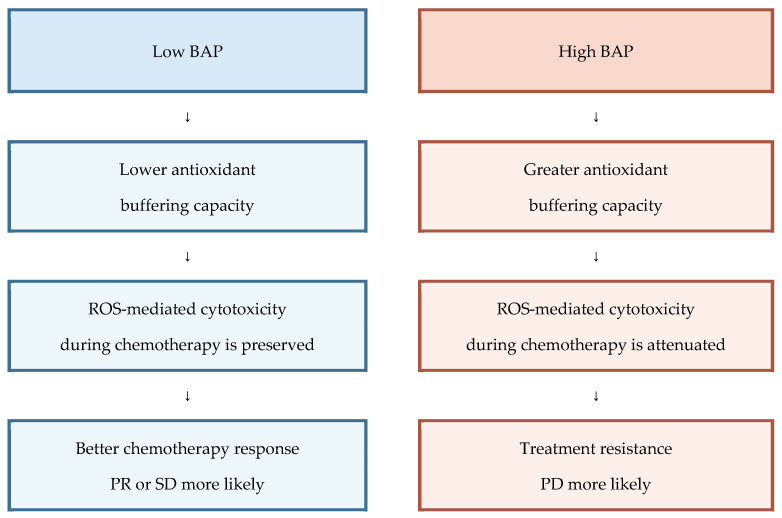
Schematic summary of the association between baseline biological antioxidant potential and chemotherapy response in patients with stage IV colorectal cancer. Low biological antioxidant potential (BAP) was associated with lower antioxidant buffering, preserved reactive oxygen species (ROS)-mediated cytotoxicity during chemotherapy, and better radiologic response. In contrast, high BAP was associated with greater antioxidant buffering, attenuated ROS-mediated cytotoxicity, progressive disease or treatment resistance. This schematic summarizes the observed cohort associations and a plausible biological interpretation. Abbreviations: BAP, biological antioxidant potential; PD, progressive disease; PR, partial response; ROS, reactive oxygen species; SD, stable disease.

Using an ordinal therapeutic effect score (PR = 1, SD = 2, and PD = 3), with higher scores indicating poorer therapeutic response, multivariate linear regression adjusted for age, sex, number of metastatic organs, number of chemotherapy regimens, and BAP level showed that higher BAP was independently associated with a worse therapeutic effect score (β = 0.282; 95% CI, 0.0001–0.0008; *p* = 0.009) (Table 3). In contrast, d-ROMs were not associated with the therapeutic effect score. To facilitate interpretation of the multivariate results, we added a schematic summary of the relationship between baseline BAP and chemotherapy response (Figure 2). This schematic illustrates the observed clinical associations and a plausible biological interpretation, without implying causality.

**Table 3:** Multivariate linear regression analysis of the therapeutic effect.

Independent Variables	Multivariate Linear Regression Analysis	
	β (95%CI)	*p*-Value
BAP	0.282 (0.0001–0.0008)	0.009

Adjusted for age, sex, tumor size, lymph node metastasis, BAP levels, number of metastatic organs, and number of chemotherapy regimens. Abbreviations: BAP, biological antioxidant potential; β, regression coefficient; CI, confidence interval.

### 3.5. DSS According to Therapeutic Response

**Figure 3:**
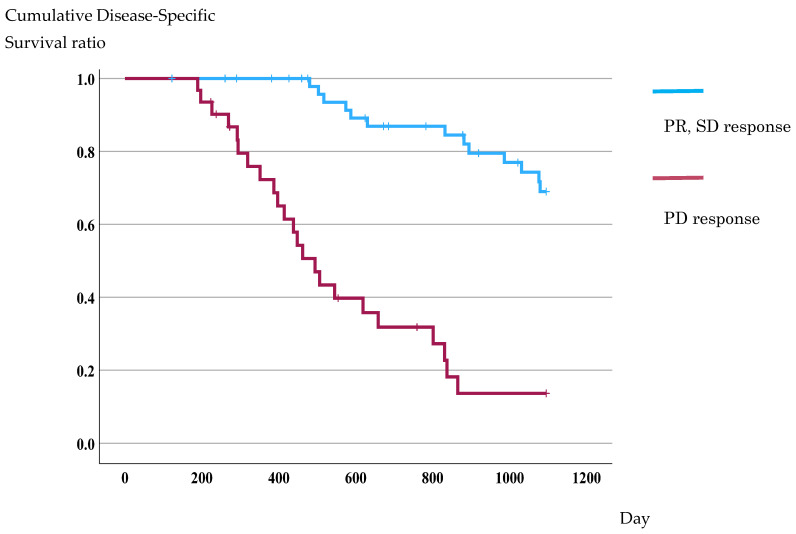
Kaplan–Meier analysis of disease-specific survival according to the therapeutic response. Patients with a good therapeutic response had a better prognosis compared with those with a poor response (69.0% vs. 13.6%; log-rank *p* < 0.001).

During the follow-up, 36 colorectal cancer-specific deaths occurred, and the 3-year disease-specific survival (DSS) for the overall cohort was 49.1%. In Kaplan–Meier analyses based on therapeutic response, patients who achieved PR or SD showed significantly better outcomes than those with PD (69.0% vs. 13.6%; log-rank *p* < 0.001) (Figure 3).

### 3.6. Multivariable Analysis of Prognosis According to Therapeutic Response

In the multivariable Cox model adjusted for age, sex, number of metastatic organs, number of chemotherapy regimens, and therapeutic response, a good therapeutic response remained an independent predictor of better DSS (HR, 7.641; 95% CI, 3.693–15.810; *p* < 0.001).

### 3.7. DSS According to Redox Biomarker Levels

**Figure 4:**
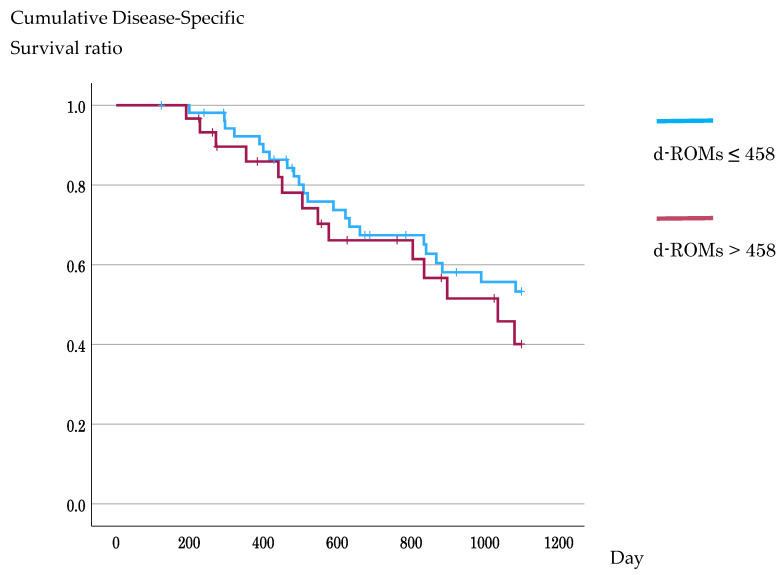
Kaplan–Meier analysis of disease-specific survival according to the d-ROM cutoff value. Patients were stratified by the receiver operating characteristic-derived d-ROM cutoff value of 458 U.CARR. No significant difference in 3-year disease-specific survival was observed between the low- and high-d-ROM groups (53.3% vs. 40.1%; log-rank *p* = 0.373). d-ROMs, derivatives of reactive oxygen metabolites.

**Figure 5:**
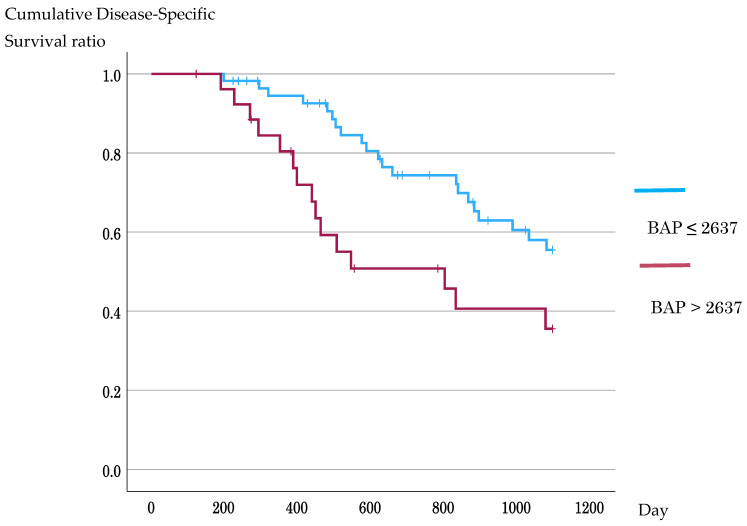
Kaplan–Meier analysis of disease-specific survival according to the BAP cutoff value. Patients were stratified by the receiver operating characteristic-derived BAP cutoff value of 2637 μmol/L. Patients with high BAP levels had significantly poorer 3-year disease-specific survival than those with low BAP levels (35.6% vs. 55.5%; log-rank *p* = 0.019). BAP, biological antioxidant potential.

In Kaplan–Meier analyses using ROC-derived cutoffs, DSS did not differ significantly between the d-ROM groups (3-year DSS, 53.3% vs. 40.1%; log-rank *p* = 0.373), whereas patients with high BAP had significantly worse DSS than did those with low BAP (3-year DSS, 35.6% vs. 55.5%; log-rank *p* = 0.019) (Figure 4 and Figure 5). In the supplementary analysis using the median BAP value of 2480 μmol/L as the cutoff, survival outcomes did not differ significantly between the low- and high-BAP groups. These results are shown in Supplementary Material (3-year DSS, 50.2% vs. 48.3%; log-rank *p* = 0.427) (Figure S3).

### 3.8. Multivariable Analysis of Prognosis According to d-ROM and BAP Levels

In the multivariable Cox model adjusted for age, sex, number of metastatic organs, number of chemotherapy regimens, and BAP levels, high BAP remained an independent predictor of poorer DSS (HR, 2.174; 95% CI, 1.103–4.283; *p* = 0.009). In contrast, d-ROM levels were not independently associated with DSS.

## 4. Discussion

Three important findings emerged from this study. First, in cases of stage IV CRC, the BAP value, an indicator of antioxidant capacity measured before primary tumor resection, was useful in predicting the therapeutic effect of postoperative chemotherapy. Second, the BAP value was also useful in predicting the three-year DSS, even after adjusting for age, sex, number of metastatic organs, and number of chemotherapy regimens. Third, d-ROM value, an indicator of oxidative stress, was not useful in predicting treatment efficacy or prognosis. These findings indicate that antioxidant buffering capacity, rather than oxidative load itself, may be a critical determinant of chemotherapy sensitivity and prognosis in metastatic colorectal cancer.

### 4.1. Mechanisms of Tumor Oxidative Stress and Antioxidant Adaptation

Cancer cells are exposed to persistently elevated oxidative stress caused by oncogenic signaling, mitochondrial dysfunction, and hypoxic conditions. To survive, tumors activate antioxidant programs such as NRF2, which controls the balance between oxidative stress and antioxidant capacity [5]. NRF2 is a central regulator of the cellular antioxidant system. Under basal conditions, NRF2 forms a complex with Kelch-like ECH-associated protein 1 (Keap1). When oxidative stress induces oxidative modification of cysteine residues in Keap1, the NRF2–Keap1 complex dissociates, and NRF2 translocates to the nucleus to activate the transcription of genes involved in antioxidant and cytoprotective responses [8,11,23]. Consistent with this program, the expression of GSH biosynthetic enzymes, thioredoxin reductase, and metabolic enzymes that support NADPH production increases. Downstream, the GSH system detoxifies ROS via GSH peroxidases and glutathione S-transferases (GSTs), while GSH reductase uses NADPH to regenerate reduced GSH. In parallel, the TRX system maintains peroxiredoxins in a reduced state, thereby enabling the rapid detoxification of peroxides. In addition, SODs and CAT limit ROS amplification [4].

### 4.2. Chemotherapy-Induced Oxidative Stress and Resistance Mechanisms

Many chemotherapeutic agents exert antitumor effects by utilizing oxidative stress as part of their mechanism of action. Oxidative and antioxidative processes are also involved in molecular targeted therapies. Bevacizumab (an anti-VEGF antibody) inhibits angiogenesis, resulting in hypoxia within tumors. Consequently, oxidative stress increases, which reportedly enhances anticancer drug efficacy. In addition, cetuximab, a monoclonal anti-epidermal growth factor receptor (EGFR) antibody, not only competitively inhibits ligand binding, such as epidermal growth factor (EGF) and transforming growth factor-alpha, to the extracellular domain of EGFR but also downregulates the EGFR–glutamine transporter alanine serine cysteine transporter 2 (ASCT2) complex. Consequently, the supply of glutamine, which is essential for the synthesis of the antioxidant GSH, is reduced, leading to decreased ROS scavenging capability [12]. Conversely, tumor cells may adapt to oxidative stress by strengthening antioxidant defenses, thereby enabling their survival and conferring resistance to anticancer drugs [23,25,34].

Studies at the cellular level have reported that NRF2 activation enhances resistance to platinum-based drugs and fluoropyrimidines in several cancers and that GSH maintenance protects cancer cells from anticancer agents [34]. Pharmacological inhibition of GSH synthesis, TRX reductase, or NRF2 signaling reportedly enhances sensitivity to chemotherapy [5,10,11,34,35]. Regulation of ROS processing pathways in CRC models has been implicated in resistance to 5-FU, suggesting that targeting these pathways may enhance drug sensitivity [23].

### 4.3. Clinical Evidence Linking Antioxidant Capacity to Treatment Response

From a clinical perspective, Baltruskeviciene et al. reported that administering cytotoxic agents, including 5-FU, epirubicin, cyclophosphamide, or paclitaxel, as adjuvant therapy increased oxidative stress in patients with breast cancer. They also reported that 3 weeks after receiving an adjuvant chemotherapy cycle, patients showed a significant decrease in serum concentrations of GSH, GST, nitric oxide, and GSH reductase [36]. Similarly, studies in patients with gastric cancer treated with 5-FU, Adriamycin, and mitomycin; colon cancer treated with 5-FU, oxaliplatin, and folinic acid; and prostate cancer treated with prednisolone and mitoxantrone have reported increased oxidative stress levels after initiating first-line chemotherapy [37]. These findings suggest that antioxidant molecules are consumed during treatment.

Several clinical studies in gastrointestinal cancers have assessed systemic antioxidant capacity, although few have evaluated pretreatment antioxidant capacity as a predictor of chemotherapy response or prognosis. In patients with CRC, Santiago-Arteche et al. reported that plasma total antioxidant capacity measured with 3-ethylbenzothiazoline-6-sulfonic acid and ferric reducing antioxidant power decreased after chemotherapy and was lower in patients with metastases, whereas Chiang et al. showed that Trolox equivalent antioxidant capacity and GSH-related antioxidant indices changed after tumor resection and chemotherapy [25,38]. In gastric cancer, Lu et al. reported that patients with increased antioxidant indices after neoadjuvant chemotherapy had better treatment responsiveness, and Du et al. showed that preoperative total oxidant/antioxidant imbalance predicted poorer survival after curative resection [39,40].

Experimental evidence further suggests that administration of antioxidants may promote tumor survival or diminish treatment effectiveness [9]. For example, administration of vitamin E in a mouse cholangiocarcinoma model induced the expression of the antioxidant enzyme heme oxygenase-1 in cancer tissue, leading to reduced chemotherapy effectiveness [22]. Similarly, among patients with tongue cancer who underwent chemoradiotherapy, those with lower antioxidant capacity before treatment exhibited better therapeutic responses [41]. Despite these findings, relatively few studies have examined the therapeutic effectiveness and antioxidant capacity in CRC. Our observation that higher BAP levels were associated with lower responsiveness to anticancer drugs is consistent with these experimental and clinical findings.

### 4.4. Clinical Significance of BAP as a Predictive Biomarker

Cysteine has been implicated in differences in antioxidant capacity in the blood and within cells. As a major substrate for intracellular GSH synthesis, serum cysteine levels are thought to correlate with intracellular antioxidant capacity. In addition, cysteine contains a thiol group (-SH), which can directly eliminate free radicals [42]. Jansen et al. reported a correlation between total thiol assay values and BAP values; although this correlation is indirect, BAP values may reflect intracellular antioxidant capacity [43]. The BAP value, which indicates antioxidant capacity measured before primary tumor resection, was also considered useful for predicting the 3-year DSS rate.

Regarding the relationship between antioxidant capacity and prognosis, Boakye et al. reported that higher antioxidant capacity is associated with a better prognosis [44]. However, these reports do not focus solely on cases treated with anticancer drugs. As the present study targeted cases in which anticancer drug treatment was administered in the presence of residual cancer, the results may differ from those of previous studies.

Antioxidant capacity reportedly changes during treatment, raising the question of which time point should be considered. Multiple studies have reported that in stage IV CRC, prognosis is better when tumor shrinkage is achieved with first-line therapy; therefore, measuring BAP at the initial stage of treatment, as performed in this study, may be useful [45,46].

We have previously reported that d-ROM values are effective for prognostic prediction. However, in this study, no association was found between d-ROMs and treatment efficacy or prediction of the 3-year DSS. Limiting the present study to stage 4 cases treated with anticancer drugs may have influenced these results. In addition, as the measurements were obtained before tumor resection, changes in oxidative stress caused by tumor removal may have influenced the findings. Furthermore, we previously reported on changes in d-ROMs and BAP associated with tumor resection [32]. One month after surgery, no resection-related changes in BAP were observed. However, d-ROM levels have been reported to decrease in correlation with tumor size, which may also have influenced the results.

### 4.5. Study Limitations and Directions for Future Research

Some limitations should be considered when interpreting the findings of this study. First, the BAP assay measures ferric reducing capacity in serum and is influenced by circulating antioxidants, including uric acid, albumin, and low-molecular-weight reducing molecules. Therefore, BAP is a non-specific systemic measure and may not directly quantify intracellular glutathione concentrations, NRF2 activity, or other tumor cell-intrinsic antioxidant defense mechanisms. Concurrent assessment of BAP with NRF2 expression or activation status, glutamate-cysteine ligase catalytic subunit, glutathione-related enzymes, and thiol metabolites could strengthen mechanistic interpretation and causal inference. Second, in the analysis using Kaplan–Meier survival curves for the 3-year DSS study and ROC analysis for determining the cutoff values of d-ROMs and BAP, the AUC values were close to 0.5, indicating that these cutoff values may be unreliable. On the other hand, this study confirmed that patients with a good therapeutic response had a favorable prognosis, indicating that BAP levels may at least indirectly be associated with prognosis. Finally, the systemic redox state may change during surgery and chemotherapy. Prospective studies with longitudinal measurements of d-ROMs and BAP throughout the treatment period are needed to clarify whether changes in oxidative stress and antioxidant capacity are associated with treatment efficacy, acquired resistance, and survival outcomes.

### 4.6. Future Perspectives

Future research should validate the prognostic relevance of BAP in larger prospective cohorts and determine whether serial changes in BAP during chemotherapy predict acquired resistance or treatment failure. Combining BAP with tumor-based markers, such as NRF2 expression, and circulating biomarkers, such as circulating tumor DNA, may improve prognostic accuracy beyond BAP alone. Such integrated approaches may help clarify whether systemic antioxidant capacity is only a surrogate marker of treatment response or is mechanistically linked to therapeutic resistance.

## 5. Conclusions

In this observational study of patients with stage IV CRC undergoing systemic chemotherapy, elevated baseline systemic antioxidant capacity was independently associated with reduced treatment effectiveness and poorer DSS. In contrast, systemic oxidative stress levels reflected the tumor burden but did not predict therapeutic response or prognosis. These findings indicate that antioxidant buffering capacity, rather than oxidative load alone, may play a more critical role in determining chemotherapy sensitivity in patients with mCRC.

Our results support the concept that excessive antioxidant defense may attenuate the ROS-mediated cytotoxic mechanisms of anticancer drugs and contribute to therapeutic resistance. Simple blood-based assessment of antioxidant capacity using standardized assays, such as BAP, may provide a feasible clinical biomarker for identifying patients at a higher risk of poor response to conventional chemotherapy.

## Figures and Tables

**Table 1:** Comparison of preoperative d-ROM and BAP values with clinicopathological factors in patients with stage IV colorectal cancer (Mann–Whitney U test).

Independent Variables	Case	d-ROMs		BAP	
		Median (P_25_–P_75_)	*p*-Value	Median (P_25_–P_75_)	*p*-Value
Age (years)			0.270		0.993
<70	51	435 (373–511)		2481 (2289–2701)	
≥70	33	425 (355–476)		2480 (2251–2696)	
Sex			0.730		0.877
Male	49	428 (373–472)		2485 (2232–2730)	
Female	35	437 (346–520)		2468 (2346–2649)	
BMI			0.989		0.635
<23	57	431 (373–490)		2532 (2289–2696)	
≥23	27	429 (366–474)		2458 (2220–2671)	
Smoking			0.19		0.489
No	39	428 (366–472)		2462 (2288–2661)	
Yes	45	458 (386–506)		2579 (2222–2762)	
Location			0.652		0.225
Right	29	436 (355–506)		2579 (2323–2737)	
Left	55	428 (371–461)		2473 (2246–2673)	
Tumor size (mm)			0.046		0.168
<50	48	401 (347–456)		2463 (2246–2665)	
≥50	36	455 (419–494)		2551 (2358–2753)	
Tumor invasion depth			0.185		0.518
T2, T3	17	397 (325–512)		2474 (2232–2623)	
T4	67	435 (376–481)		2485 (2288–2734)	
Lymph node metastasis			0.806		0.094
No	18	423 (350–494)		2600 (2467–2769)	
Yes	66	433 (373–481)		2460 (2263–2695)	
Number of metastatic organs			0.496		0.685
Single-organ metastasis	54	436 (367–511)		2536 (2260–2703)	
Multiple-organ metastasis	30	427 (377–446)		2460 (2288–2678)	
Preoperative chemotherapy			0.678		0.627
No	72	432 (366–494)		2481 (2288–2681)	
Yes	12	422 (373–461)		2582 (2213–2837)	

Abbreviations: BMI, body mass index; d-ROMs, derivatives of reactive oxygen metabolites; BAP, biological antioxidant potential; P_25_–P_75_, 25th–75th percentile (interquartile range).

**Table 2:** Histological responses and clinical factors.

	Therapeutic Effect		*p*-Value
	PR + SD (53 Cases)	PD (31 Cases)	
Age (years)			0.934
<70	32	19	
≥70	21	12	
Sex			0.379
Male	29	20	
Female	24	11	
Tumor size (mm)			0.300
Median (P_25_–P_75_)	50 (40.0–60.0)	45 (36.6–58.5)	
Tumor invasion depth			0.125
T2, T3	8	9	
T4	45	22	
Lymph node metastasis			0.723
No	12	6	
Yes	41	25	
Number of metastatic organs			0.167
Single-organ metastasis	37	17	
Multiple-organ metastasis	16	14	
chemotherapy regimens (postoperative chemotherapy)			0.896
oral 5-FU agent	6	4	
doublet regimen	25	13	
Add molecular targeted therapy to the doublet regimen	22	14	
d-ROMs			0.594
Median (P_25_–P_75_)	429 (368–472)	437 (371–506)	
BAP			0.003
Median (P_25_–P_75_)	2446 (2232–2621)	2655 (2434–2822)	
CEA			0.499
Median (P_25_–P_75_)	10.3 (3.6–51.4)	7.8 (3.45–28.2)	
Preoperative chemotherapy			0.782
No	45	27	
Yes	8	4	

Abbreviations: PR, partial response; SD, stable disease; PD, progressive disease; P_25_–P_75_, 25th–75th percentile (interquartile range); d-ROMs, derivatives of reactive oxygen metabolites; BAP, biological antioxidant potential; CEA, carcinoembryonic antigen. Continuous variables, including d-ROMs, BAP, and CEA, were compared using the Mann–Whitney U test.

## Data Availability

The datasets generated and/or analyzed during the current study are not publicly available due to privacy and ethical restrictions.

## Supplementary Materials

The following supporting information can be downloaded at https://www.mdpi.com/article/10.3390/antiox15050595/s1, Figure S1: ROC curve for d-ROMs to predict DSS; Figure S2: ROC curve for BAP to predict DSS; Figure S3: Kaplan-Meier survival curves according to the median BAP cutoff; Table S1: Chemotherapy regimens and targeted agents used in the study cohort.

## Abbreviations

The following abbreviations are used in this manuscript:

ASCT2	alanine serine cysteine transporter 2
AUC	area under the curve
BAP	biological antioxidant potential
BMI	body mass index
CAT	catalase
CEA	carcinoembryonic antigen
CI	confidence interval
CRC	colorectal cancer
CT	computed tomography
d-ROMs	derivatives of reactive oxygen metabolites
DSS	disease-specific survival
EGF	epidermal growth factor
EGFR	epidermal growth factor receptor
GSH	glutathione
GST	glutathione S-transferase
HR	hazard ratio
NRF2	nuclear factor erythroid 2-related factor 2
PD	progressive disease
PR	partial response
RECIST	Response Evaluation Criteria in Solid Tumors
ROC	receiver operating characteristic
ROS	reactive oxygen species
SD	stable disease
SOD	superoxide dismutase
VEGF	vascular endothelial growth factor
5-FU	5-fluorouracil
