# Supplementary material for: Serum Antioxidant Capacity Predicts Prognosis in Patients with Metastatic Colorectal Cancer: An Original Cohort Study
Source: Antioxidants (Basel). 2026 May 8;15(5):595. doi: 10.3390/antiox15050595 (PMC13203352; doi:10.3390/antiox15050595)
Supplement: Supplementary file 1 [file antioxidants-15-00595-s001.zip › antioxidants-4255796-supplementary.pdf]

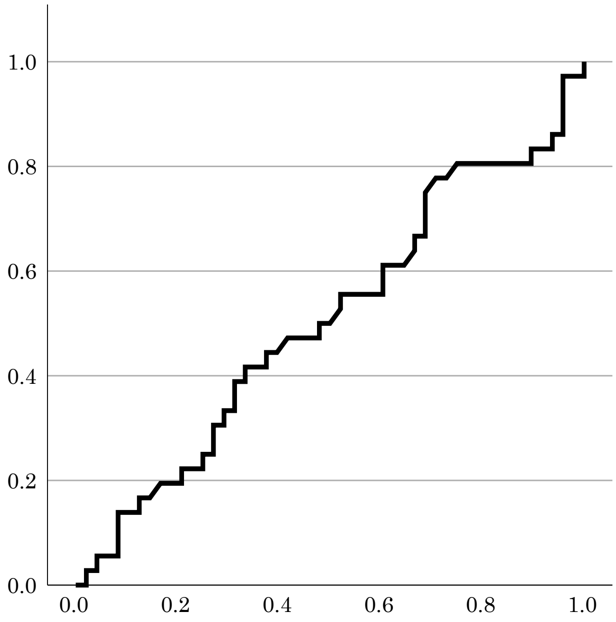

Figure S1. ROC curve for d-ROMs to predict DSS.
Area under the curve = 0.559.

ROC, receiver operating characteristic; d-ROMs, derivatives of reactive oxy- gen metabolites; DSS, disease-specific survival

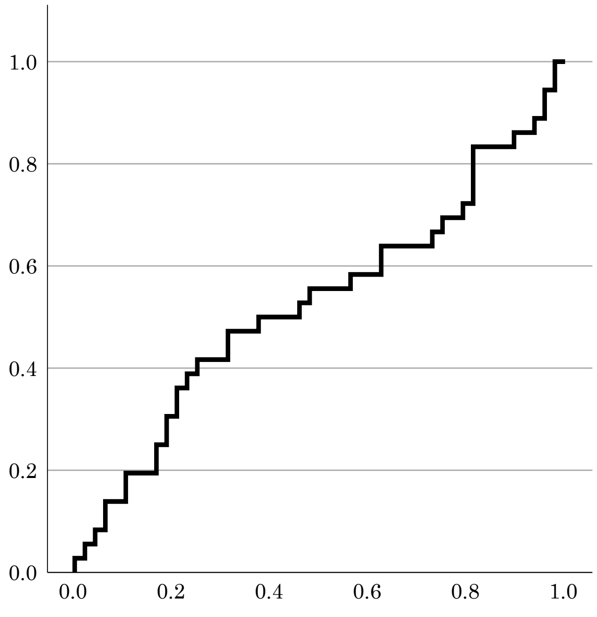

Figure S2. ROC curve for BAP to predict DSS.
Area under the curve = 0.509.

ROC, receiver operating characteristic; BAP, biological antioxidant potential;
DSS, disease-specific survival

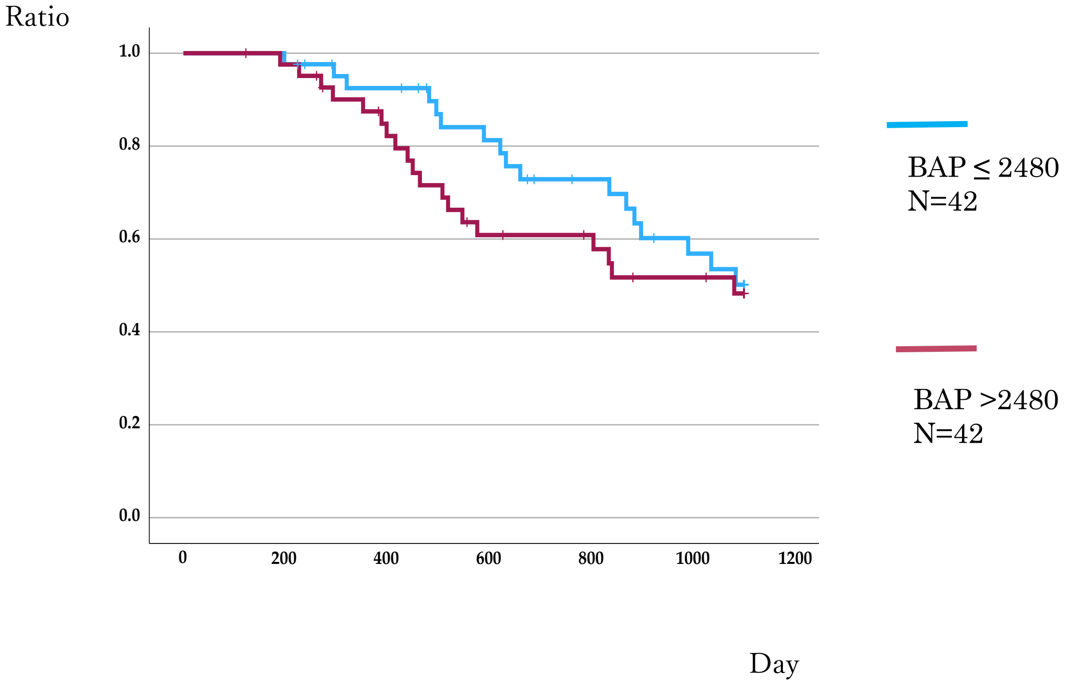

Figure S3. Kaplan-Meier survival curves according to the median BAP cutoff.

Patients were classified into low- and high-BAP groups using the median BAP value of 2480 μmol/L as the cutoff. No significant difference in survival was observed between the two groups (3-year DSS, 50.2% vs. 48.3%; log-rank P=0.427).

BAP, biological antioxidant potential; DSS, disease-specific survival

Supplementary Table S1. Chemotherapy regimens and targeted agents used in the study cohort

Treatment category	Chemotherapy regimen	Targeted agent	Number of patients
Oral fluoropyrimidine	UFT	None	2
	Capecitabine	None	8
Cytotoxic doublet	mFOLFOX6	None	21
	CAPEOX	None	16
	SOX	None	1
Doublet plus targeted agent	mFOLFOX6	Panitumumab	14
	mFOLFOX6	Bevacizumab	12
	CAPEOX	Bevacizumab	8
	FOLFIRI	Bevacizumab	1
	FOLFIRI	Ramucirumab	1

Abbreviations: UFT, tegafur/uracil; mFOLFOX6, modified fluorouracil, leucovorin, and oxaliplatin; CAPEOX, capecitabine plus oxaliplatin; SOX, S-1 plus oxaliplatin; FOLFIRI, fluorouracil, leucovorin, and irinotecan.
